# Differentiation of sarcoidosis-lymphoma syndrome lesions: a case report on the use of two different positron emission tomography tracers

**DOI:** 10.1186/s12880-015-0104-x

**Published:** 2016-01-08

**Authors:** Ryan Yudistiro, Yukiko Arisaka, Azusa Tokue, Takahito Nakajima

**Affiliations:** Department of Diagnostic Radiology and Nuclear Medicine, Gunma University Graduate School of Medicine, 3-39-22 Showa, Maebashi, 371-8511 Gunma Japan; Department of Diagnostic Radiology and Nuclear Medicine, Gunma University Hospital, Gunma, Japan

**Keywords:** FDG, Amino acid tracer, Sarcoidosis–lymphoma syndrome, Positron emission tomography (PET), LAT1

## Abstract

**Background:**

Sarcoidosis–lymphoma syndrome (SLS) is a rare disease in which both entities coexist. We aimed to study the role of ^18^F-fluorodeoxyglucose (FDG) and L–[3-^18^F] α-methyltyrosine (FAMT) positron emission tomography (PET)/computed tomography (CT) in differentiating between these two lesions.

**Case presentation:**

A 54-year-old female with large liver tumors was referred to our Nuclear Medicine Department for staging using FDG PET/CT. She had a history of primary biliary cirrhosis (PBC) for 15 years and developed lung and mediastinal sarcoidosis 1 year before the liver tumors were noted. Abdominal dynamic CT revealed two well-circumscribed, peripherally-enhancing, low-density masses in the right lobe of the liver with intensive ring-form FDG uptakes at maximum standard uptake values (SUVmax) of 18.3 and 19.5, respectively. In the arterial phase, a hepatic artery was seen penetrating the tumor, a phenomenon known as “angiogram sign”. Chest PET/CT findings showed irregular thickening of the bronchovascular bundles, central peribronchial shaggy consolidations in the right middle and lower lobes (SUVmax, 4.6), and mediastinal and hilar lymphadenopathies (SUVmax, 2.7).

After assessment, chemotherapy with rituximab, cyclophosphamide, doxorubicin hydrochloride, vincristine sulfate, and prednisone (R–CHOP) was administered for eight cycles. Follow-up imaging studies using FDG and FAMT PET/CT were performed 3 months after the last cycle of chemotherapy, which showed that the two highly FDG-avid tumors in the liver had disappeared. However, faint FDG uptake persisted in the lung consolidations (SUVmax, 6.3), and FDG uptake for the mediastinal lymphadenopathies increased (SUVmax of 5.8). In contrast, there was no significant uptake of FAMT in the liver, as well as in the lungs and the bilateral mediastinal lymphadenopathies. These discrepant uptakes between FDG and FAMT were compatible with sarcoidosis.

**Conclusion:**

Combination of FDG and FAMT in PET/CT studies may play an important role in the management of SLS patients, especially in differentiating between sarcoidosis and lymphoma lesions.

## Background

Sarcoidosis is defined as a multisystem granulomatous disorder of unknown cause. It frequently presents in young and middle-aged adults with pulmonary infiltrates and bilateral hilar and mediastinal lymphadenopathies, but it may rarely affect the eyes, liver, spleen, kidneys, and heart [1]. The annual incidence of sarcoidosis is 15–40 per 100,000 of the population [2]. Chronic inflammation is a putative mediator of the risk for sarcoidosis, and concomitant malignancy is found in 1.2–2.5 % [2, 3]. The main types of malignancies that may occur with sarcoidosis are lung cancer, lymphoma, testicular cancer, and uterine cancer [4].

Since positron emission tomography (PET) with ^18^F-fluorodeoxyglucose (FDG) is very sensitive for detecting malignant lesions, it is often used to evaluate the extent of malignancies. However, FDG is not only accumulated in malignant cells but also in granulomatous lesions, such as sarcoidosis, making it difficult to differentiate between the two lesions [2, 5, 6]. L-[3-^18^F]-α-methyltyrosine (FAMT) is an amino acid analog PET tracer that reversibly accumulates in cells through L-type amino acid transporter 1 (LAT1). FAMT was reported to have high accumulation in malignant lesions, but not in benign lesions [2].

In this study, we reported the coexistence of sarcoidosis and lymphoma, or sarcoidosis-lymphoma syndrome (SLS), and demonstrated the role of FDG and FAMT in PET/computed tomography (CT) study for differentiating between these two lesions.

## Case presentation

A 54-year-old female was referred to our Nuclear Medicine Department for FDG PET/CT staging of large liver tumors that were incidentally detected by abdominal ultrasonography during her routine follow-up for primary biliary cirrhosis (PBC) in another hospital. She had a history of PBC for 15 years and developed lung and mediastinal sarcoidosis 1 year before the liver tumors were noted. Sarcoidosis was diagnosed by the clinical features; uveitis and high level of angiotensin converting enzyme (22.2 IU/L; normal range, 8.3–21.4 IU/L), and the biopsy specimens from lung consolidation and pleura. Long-term steroid therapy was not administered because it was contraindicated for cirrhosis. She only received oral ursodeoxycholic acid 600 mg/day for cirrhosis.

On examination, she was noted to be in a stable and good condition; despite increased serum levels of lactate dehydrogenase (LDH), interleukin-2 receptor (IL-2R), γ-guanosine-5′-triphosphate (GTP), alkaline phosphatase (ALP), and C-reactive protein (CRP), white blood cell count and liver function tests [alanine transaminase (ALT) and aspartate transaminase (AST)] were still within normal limits.

**Fig. 1:**
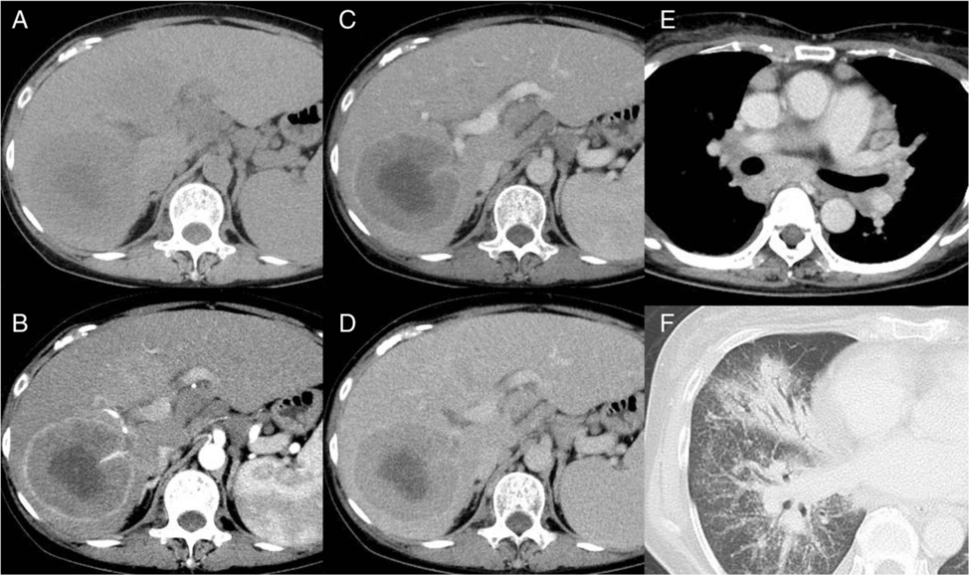
Abdominal dynamic CT images in a 54-years old female with sarcoidosis–lymphoma syndrome. **a**-**d** Plain CT image shows two well-circumscribed, peripherally-enhancing, low-density masses in the anterior segment (S5; 80 × 78 × 88 mm) and inferior segment (S7; 62 × 88 × 82 mm) of the right lobe of the liver. In the arterial phase, a hepatic artery is seen penetrating inside the tumor. *CT* computed tomography. Chest CT images in a 54-years old female with sarcoidosis-lymphoma syndrome. **e** Contrast enhancement shows mediastinal and hilar lymphadenopathies. **f** On lung window, there is irregular thickening of the bronchovascular bundles and central peribronchial fibro-infiltration lesions in the right middle and lower lobes

On abdominal dynamic CT (Fig. 1), two well-circumscribed, peripherally-enhancing, low-density masses were seen in the anterior segment (S5; 80 × 78 × 88 mm) and inferior segment (S7; 62 × 88 × 82 mm) of the right lobe of the liver. In the arterial phase, a hepatic artery was seen penetrating the tumor, a phenomenon known as “angiogram sign”, which can be seen in less invasive tumors, such as lymphoma. The hepatosplenomegaly that was noted was attributed to liver cirrhosis due to PBC. Chest CT findings showed irregular thickening of the bronchovascular bundles, central peribronchial shaggy consolidations in the right middle and lower lobes, and mediastinal and hilar lymphadenopathies. These findings were considered as lymphoma; however, ruling out sarcoidosis remained difficult.

PET/CT images were acquired on a PET/CT scanner (a Discovery STE, GE Healthcare, USA). At the time of FDG injection, this patient had fasted for more than 6 h and had blood sugar levels of 114 mg/dL (6.3 mmol/L). The scans were performed at 1 h after intravenous injection of either FDG or FAMT at the dose of 5.0 MBq/kg. Patient was scanned from the thigh to the head in the arms-down position with 700-mm field of view (FOV) and a slice thickness of 3.27 mm. Three- dimensional (3D) data acquisition was performed for 3 min per bed position, followed by image reconstruction with the 3D-ordered-subsets expectation maximization method. Segmented attenuation was corrected by X-ray CT (140 kV, 120–240 mAs) to produce 128 × 128 matrix images. CT images were reconstructed using a conventional filtered back projection method.

**Fig. 2:**
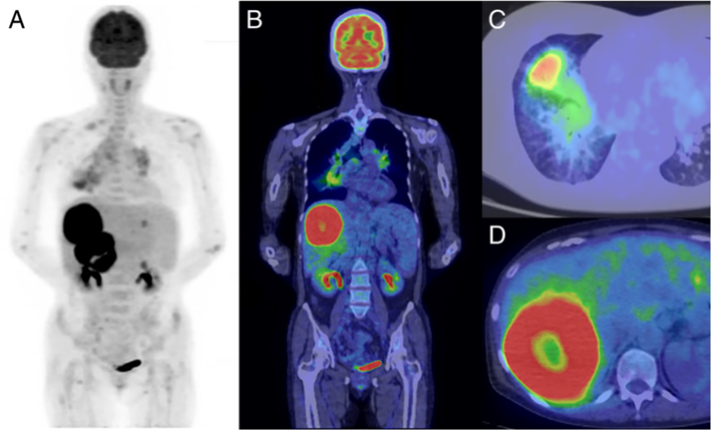
Pre-therapy FDG PET/CT images in a 54-year-old female with sarcoidosis–lymphoma syndrome. Whole-body scan of FDG PET/CT was performed at 60 min after 290.7 MBq of FDG injection. (**a** MIP; **b**. coronal fusion; **c**-**d**. axial fusion) In the liver, there were intensive ring-form uptakes in the tumors, with maximum standard uptake values (SUVmax) of 18.3 in S5 and 19.5 in S7. The faint right middle and lower lobe consolidations had moderate FDG uptake (SUVmax, 4.6), whereas the multiple bilateral hilar and mediastinal lymphadenopathies showed mild FDG uptake (SUVmax, 2.7). *FDG* 18-fluorodeoxyglucose, *PET* positive emission tomography, *CT* computed tomography, *MIP* maximum intensity projection

FDG PET/CT images (Fig. 2) showed intensive ring-form FDG uptakes of the two liver tumors, with maximum standard uptake values (SUVmax) of 18.3 in S5 and 19.5 in S7. Biopsy of these liver tumors was done. The faint right middle and lower lobe consolidations had moderate FDG uptake (SUVmax 4.6), whereas the multiple bilateral hilar and mediastinal lymphadenopathies showed mild FDG uptake (SUVmax 2.7).

**Fig. 3:**
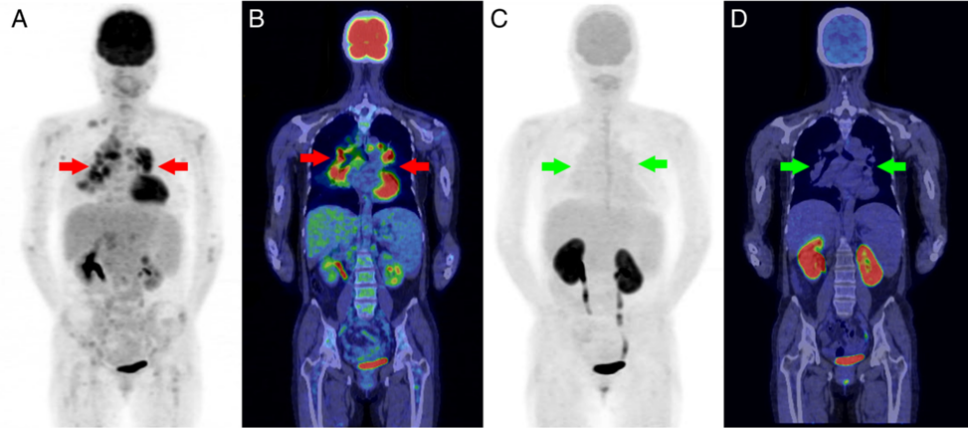
PET/CT images in a 54-year-old female with sarcoidosis–lymphoma syndrome after chemotherapy. Whole-body scan of FAMT PET/CT was performed at 60 min after 281.7 MBq of FAMT injection. (**a**-**b** FDG PET/CT; **c**-**d**. FAMT PET/CT). High FDG uptake in the mediastinal lymphadenopathies (*red arrow*) was still seen. The FDG–avid liver tumor lesions disappeared. No FAMT uptake that was compatible with sarcoidosis lesions was seen in the mediastinal lymphadenopathies (*green arrow*). *FDG* ^18^F-fluorodeoxyglucose, *PET* positive emission tomography, *CT* computed tomography, *FAMT* L–[3-^18^F] α-methyltyrosine

After the pre-therapy assessment, chemotherapy with rituximab, cyclophosphamide, doxorubicin hydrochloride, vincristine sulfate, and prednisone (R–CHOP) was administered for eight cycles. Prednisone was administered as part of R-CHOP in very short time that apparently less effective for sarcoidosis treatment. Follow-up 3 months after the last cycle of chemotherapy was done using FDG and FAMT PET/CT studies. The two highly FDG-avid tumors in the right lobe of the liver disappeared (Fig. 2a-b). However, there were increasing FDG uptakes in the lung consolidations (SUVmax, from 4.6 to 6.3) and bilateral mediastinal and hilar lymphadenopathies (SUVmax, from 2.7 to 5.8). On FAMT PET/CT (Fig. 2c-d), there was no significant uptake of FAMT in the liver, as well as in the lungs and mediastinum. These discrepant findings between FDG and FAMT were compatible with sarcoidosis as the etiology of the thoracic lesions (Fig. 3).

Histological examination of liver biopsy specimens obtained pre-treatment revealed large cells with round-to-irregular vesicular nuclei, variably prominent nucleoli, and scant-to-moderate amounts of cytoplasm. On immunohistochemical analysis, the tumors stained positive for CD20 and CD79a, but negative for CD3, CD5, and CD10. Therefore, the liver tumors were diagnosed as malignant diffuse large B-cell lymphoma (DLBCL).

### Discussion

Sarcoidosis appears to be associated with significantly increased risk for cancer in affected organs, particularly lung cancer and malignant lymphomas. Chronic inflammation is a putative mediator of this risk [3]. Sarcoidosis that occurs with lymphoma, or SLS, was first reported by Brincker in 1986 [7]. The etiology of SLS was hypothesized to be due to immunologic abnormalities that can occur in lymphoproliferative disease and other malignancies. Sarcoidosis and lymphoma may occur synchronously, but the onset of sarcoidosis usually precedes lymphoma by at least 1–2 years [4]. For lung cancer and non-Hodgkin’s lymphoma, the relative risk was doubled during the first decade of sarcoidosis follow-up and a 1.4-fold risk was also found for liver cancer [3].

FDG PET is a sensitive method for staging of several malignancies because of its Warburg effect. FDG is transported into the cell by glucose transporter 1 (GLUT1) and phosphorylated by hexokinase enzyme to FDG-6-phosphate, where it is trapped without being further metabolized. Aside from malignancy, infected and inflammatory tissues also accumulate FDG; this makes FDG PET less specific. Specificity of FDG PET may be increased when it is fused with CT images [5, 6].

Recently, the use of amino acid analogue radiotracers, such as ^11^C-methionine (MET) and ^18^F-FAMT in malignancy has been developed and clinically established [8, 9]. Accumulation of amino acid radiotracers in malignant tissues is thought to be due to increased amino acid metabolism, e.g., enhanced amino acid active transport. MET enters the metabolic process of RNA and protein synthesis, but FAMT in tumor cells is not incorporated into protein/RNA synthesis. Several clinical trials on the evaluation of sarcoid lymphadenopathy using MET and FAMT in comparison with FDG have been published [2]. It is likely that high FDG accumulation in sarcoidosis lesions was due to abundant presence of inflammatory cells and granulomas. On the other hand, accumulation of MET was considered to be low because MET uptake in inflammation has been reportedly low. Good prognosis may be expected for sarcoidosis lesions with high FDG uptake, but not for those with MET uptake [8].

Therefore, FAMT has been developed as an amino acid tracer for PET imaging and its potential use for detecting neoplasm has been confirmed on experimental tumor models [10]. FAMT is accumulated in tumor cells through LAT1, without further metabolism. A study by Kaira et.al, on sarcoidosis lesion showed high FDG uptake, but no significant FAMT uptake [2]. However these results do not suggest that FAMT will replace FDG for the diagnosis of sarcoidosis; rather, it may be useful to combine FDG and FAMT for differentiating sarcoidosis and malignant lesions [2].

In our case, FDG PET/CT played an important role in the staging, identification of appropriate sites for biopsy, assessment of response to therapy, and detection of recurrent primary hepatic lymphoma. Although FDG PET/CT can evaluate disease activity of mediastinal lymphadenopathies and lung lesions due to sarcoidosis, differentiating these lesions from malignant lymphoma remains difficult. Therefore, combining FAMT and FDG during post-therapy PET/CT studies may enable differentiation between sarcoidosis and suspicious residual lymphoma lesions [2, 5].

In the clinical study that was performed by Christian et.al in 2006 who tried to assess the clinical benefit of combined FDG PET/CT in patients with malignant lymphoma as compared to separately performed PET and CT, showed that PET was superior to CT alone. However no significance difference was observed between PET/CT and separate PET and CT imaging in patients with lymphoma. Using region-based evaluation they found the sensitivity and specificity value for CT was 85 and 91 %, for PET was 98 and 99 %, and for PET/CT was 98 and 99 % [11]. The CT presentation of primary hepatic lymphoma differs from the secondary hepatic involvement lymphoma. The secondary hepatic lymphoma is often diffusely infiltrative and difficult to detect on CT, meanwhile the primary hepatic lymphoma are easy identified on CT even before the administration of IV contrast material. The characteristic finding of primary hepatic lymphoma on CT including large poorly defined hypodense mass with satellite nodules and contrast enhancement, necrosis, and calcification appearance [12]. High uptake of FDG PET in malignant primary liver tumors in particular HCC has less sensitivity, reported range of 50–65 %, meanwhile for intrahepatic cholangiocarcinoma was more than 90 %. For lymphoma, have also been reported to show increased FDG uptakes [13].

One limitation of this case report was that FAMT PET/CT was not performed before therapy for lymphoma because evaluation of the liver tumors for a possible sarcoidosis etiology was the priority at that time. We considered no recurrence of lymphoma and persistent sarcoidosis in this case based on clinical assessment and correlation with follow-up FDG PET study, which showed persistent uptake in the thoracic lesions, but not in the liver. To clarify the usefulness of this imaging combination for SLS patients, further studies are needed to explore the role of FDG and FAMT PET/CT.

## Conclusion

Combination of FDG and FAMT in PET/CT studies may play an important role in the management of SLS patients, especially in differentiating between sarcoidosis and lymphoma lesions.

## Consent

The written informed consent was obtained before each examination was performed and for this case report to be published.

## Abbreviations

SLS: sarcoidosis–lymphoma syndrome
FDG: ^18^F-fluorodeoxyglucose
FAMT: L–[3-^18^F] α-methyltyrosine
PET: positron emission tomography
CT: computed tomography
PBC: primary biliary cirrhosis
SUVmax: maximum standard uptake value
R–CHOP: chemotherapy with rituximab, cyclophosphamide, doxorubicin hydrochloride, vincristine sulfate, and prednisone
LAT1: L-type amino acid transporter 1
LDH: lactate dehydrogenase
IL-2R: interleukin-2 receptor
GTP: γ-guanosine-5′-triphosphate
ALP: alkaline phosphatase
CRP: C-reactive protein
ALT: alanine transaminase
AST: aspartate transaminase
DLBCL: diffuse large cell B-cell lymphoma
GLUT1: glucose transporter 1
MET: ^11^C-methionine
